# User evaluation and feasibility test of an app designed for smoking cessation in Italian people who smoke: preliminary findings from an uncontrolled pre-test post-test open study

**DOI:** 10.1186/s40359-023-01430-w

**Published:** 2023-11-10

**Authors:** Pasquale Caponnetto, Mirko Casu, David Crane, Louise Ross, Maria Catena Quattropani, Riccardo Polosa

**Affiliations:** 1 Department of Educational Sciences, Section of Psychology, University of Catania, Catania, Italy; 2 Center of Excellence for the Acceleration of Harm Reduction (CoEHAR), University of Catania, Catania, Italy; 3 Department of Mathematics and Computer Science, University of Catania, Catania, Italy; 4 Research Department of Clinical, Educational and Health Psychology, University College London, London, UK; 5 Smoke Free (23 Limited), London, UK; 6 Department of Clinical & Experimental Medicine, University of Catania, Catania, Italy

**Keywords:** Smoke free, Smoking cessation, Feasibility evaluation, Addiction, mhealth, Clinical psychology

## Abstract

**Background:**

mHealth is a public health practice that exploits the use of mobile devices, including smartphone applications. We will describe an uncontrolled pre-test post-test open pilot study concerning the feasibility evaluation of a smartphone App designed to help in smoking cessation. The aim of this study is to evaluate the feasibility of a smartphone app as a tool for smoking cessation. This study is necessary to the literature because smoking is a major public health concern and has been linked to various health issues such as cardiovascular disease, respiratory disease, and cancer. While there are several smoking cessation interventions available, the use of mobile devices to aid in smoking cessation is a relatively new and innovative approach that requires further investigation.

**Methods:**

The App “Smoke Free” was configured on the devices of N = 30 participants who smoked combustible cigarette, 13 males and 17 females aged 18 to 55 years, with the indications to use it for 90 days, describe their experience, suggest new features, and report any critical aspect. The study consisted of an initial screening visit to select participants that reflected the inclusion criteria and 4 study visits: a baseline visit, two follow-up visits, and one final visit. We used descriptive stats to summarize results. Repeated measures ANOVA and Wilcoxon test were used to test differences in smoking consumption, self-reported craving, and measured eCO level. Statistical software Jamovi was used for analysis. Interviews were conducted via phone or in-person and analyzed using qualitative description principles.

**Results:**

Participants evaluated the app as having good aesthetic appeal and user-friendliness but being moderately useful, despite some quitting or reducing their smoking behavior. To improve it, participants have proposed features such as more notifications, social network integration, and damage caused by smoking to the body over time for future app updates.

**Conclusions:**

The application was moderately useful with good feasibility, with several suggestions for future updates that could improve its effectiveness.

**Supplementary Information:**

The online version contains supplementary material available at 10.1186/s40359-023-01430-w.

## Introduction

The World Health Organization (WHO) described Mobile Health (mHealth) in its 2011 report as the utilization of mobile and wireless technologies to facilitate the attainment of health objectives, with the potential to revolutionize healthcare delivery globally [1]. This approach encompasses the use of mobile devices like cell phones, patient monitoring devices, personal digital assistants, and other wireless devices, supporting medical and public health practices [1].

mHealth systems have been employed in interventions aimed at assisting individuals in their journey to quit smoking, known as mobile phone-based smoking cessation support or mCessation interventions [2]. A review conducted by Whittaker et al. [2] emphasized that the use of automated text message systems delivered to mobile phones resulted in higher cessation rates compared to the minimum standard for smoking cessation support. Similarly, Deutsch et al. [3] described a comparable system called Test My Quit (TMQ), which also relied on sending text messages to mobile phones and demonstrated its effectiveness. Liao et al. [4] conducted a text message-based smoking cessation intervention among the Chinese population and observed positive effects in a limited number of participants, indicating potential efficacy in larger samples. These types of interventions were also examined with representative samples of minority populations, yielding either positive prospects [5] or minimal impact [6].

Smartphone applications have emerged as another form of mHealth intervention for supporting smoking cessation. In a review conducted by Regmi et al. [7], eight studies examining smartphone applications designed for smoking cessation were analyzed. The use of such applications demonstrated an increase in cessation rates among smokers, although adherence to the application’s internal features appeared to influence the rates. The most favored and utilized application features were audiovisual elements, quit planning, progress tracking, and sharing features. However, inconsistent associations were observed between these features and abstinence or termination rates. Regmi and colleagues suggested conducting larger-scale studies to obtain a clearer understanding of the intervention’s effectiveness. Pbert et al. [8] compared different interventions for smoking cessation, including two smartphone applications specifically designed for quitting smoking based on mindfulness training: Craving to Quit (C2Q) and QuitSTART. The third intervention involved the use of written material. The authors found that a higher engagement with the C2Q App among heavy smokers was associated with a significant reduction in the number of cigarettes smoked compared to other conditions. Garrison et al. [9] conducted a randomized controlled experiment using the Craving to Quit App, focusing on mindfulness training. Although the results did not show a reduction in smoking rates compared to the control group, valuable data suggested that this system might weaken the association between craving and smoking, which could be beneficial in long-term cessation programs. Another application, Quit2Heal, was studied by Bricker et al. [10] in comparison with the QuitGuide from the US National Cancer Institute, specifically among cancer patients. The Quit2Heal application demonstrated promising acceptability and efficacy in helping cancer patients quit smoking.

In the forthcoming paragraphs, we will present a study that aimed to assess the feasibility of a smartphone application called Smoke Free in assisting motivated Italian smokers aiming to quit smoking. Its objective is to evaluate the app’s acceptability, usability, effectiveness in smoking cessation, and perceived usefulness within this demographic. Specifically, the Smoke Free app was installed and configured on the devices of thirty participants, who were instructed to utilize and explore its features for a duration of approximately 90 days. This study holds significance for several reasons. Firstly, smoking cessation is a matter of great public health importance, and developing effective interventions to support individuals in quitting smoking is a crucial step towards enhancing public health outcomes. To the best of our knowledge, this is the first study to evaluate the feasibility of the Smoke Free App within an Italian sample. Secondly, testing the Smoke Free app on an Italian sample is crucial as cultural, linguistic, and contextual factors can influence the app’s effectiveness and feasibility. It is important to emphasize that the application is in English, so the sample is required to use an interface in a language different from their own.

## Methods

### Study design

This study was an uncontrolled pre-test post-test open feasibility pilot study that had access to the app “Smoke Free” for three months, to test if the App would have been acceptable, effective, and useful for participants. In brief, the study consisted of an initial screening visit to select participants that reflected the inclusion criteria and 4 study visits: a baseline visit, two follow-up visits, and one final visit.

The population under study in this article is individuals who smoke combustible cigarettes. To be eligible for the study, participants had to meet specific criteria, including being 18 years or older, having smoked at least five cigarettes per day for one year or more, and being motivated to quit smoking. This criterion ensured that participants had a consistent smoking habit, which would provide a meaningful baseline for the study. Moreover, participants needed to have a smartphone, and have a fair degree of English proficiency.

The eligible sample consisted of 30 people who met the criteria mentioned above. Smoking status was verified through exhaled CO measurement (exhaled CO ≥ 6 ppm). Participants who completed the study were considered full participants, while those who dropped out or were withdrawn were considered partial participants.

At the smoking cessation clinic of the University of Catania, researchers completed the screening and baseline visit, and participants were provided with personal App codes, then downloaded the App and entered the requested information including their age, gender, number of cigarettes smoked per day and use or not of combustion-free technologies for nicotine delivery such as e-cigarettes (ECs) and heated tobacco products (HTPs). These data were securely stored on the dedicated eCRF and dashboard system.

The study was conducted according to the guidelines of the Declaration of Helsinki. Participants were recruited voluntarily, informed consents were received online before respondents answered the questionnaire, and data confidentiality was ensured according to the principles of the General Data Protection Regulation (GDPR). The study was approved by the Internal Ethic Review Board of Psychology Research – IERB on the 7th of September 2021 (Prot. n° Ierb-Edunict-2021.07.19/3) and authorized by the Ethics Committee, having the project complied with all the indications foreseen by the guidelines of the AIP (Italian Association of Psychology) and those of this Ethics Board. Written consent was obtained from participants and confidentiality and anonymity were assured.

### Study setting, recruitment, and participants

Participants were enrolled by the CoEHAR team (Center of Excellence for the acceleration of Harm Reduction) at the University of Catania. Convenience sampling was employed as individuals were recruited through advertising campaigns on social media platforms (such as Instagram and Facebook) and through word-of-mouth.

Participants who responded positively to the campaigns and were interested in the study then participated in an individual semi-structured interview, held over the phone, by email, or via instant messaging systems, during which the screening visit was carried out to verify that all the participant’s data were in line with the requirements of the study: participants were asked when and if they would have been available to use the App for three months and to have a baseline visit within 7 days, during which they received a complete description of the App usage and study procedures; smoking history and motivation to quit smoking were also evaluated.

Participants were provided written information about the project, details that it was voluntary to participate, and that confidentiality would be ensured.

### The Smoke Free app

The primary instrument used in this study is the “Smoke Free” app, which is designed to help and support people who smoke on a smoking cessation path and is available on smartphones and smartwatches. The App consists of several important sections: (a) dedicated support, which allows the user to contact a chatbot specialist in case of need; (b) a section in which to add and monitor craving states (strong desire towards smoking); (c) a Missions section, which exploits the rewarding reinforcement with a goal system that, once reached, show a reward badge to the user; (d) the main Dashboard, where you can find graphs of health improvements and amount of money saved; (e) The Diary, fundamental section in which to daily write down the details of the cessation path (e.g., number of cigarettes smoked, possible craving).

### Procedures and data collection

Individual semi-structured interviews with each participant included ten questions at the initial screening visit, four questions at the baseline visit, and twenty questions at the final visit. A recurring question concerned the number of cigarettes smoked per day. To minimize the risk of responses conditioned by bias, participants were assured that the information would be used to help better understand their smoking habits and not to judge or criticize them; moreover, it was important to show patience and empathy and understand that one of the limitations would have been remembering the exact number of cigarettes smoked, so there may have been approximations; the social desirability bias, which could have led patients to claim to smoke fewer cigarettes than they actually smoked, was minimized thanks to the recruitment requirement of “motivation to quit”. During the interviews, we also applied the “Think-Aloud” method.

Screening visit (also referred to as Visit 0): information was obtained regarding personal, demographic, and tobacco use data for each participant (for example, they were asked to judge their craving for cigarettes on a scale of 0 to 10). The use of the App was continuously monitored through the analysis based internally on the App, which shows rewards in the form of badges for each achievement of the user, and by the participants themselves in an autonomous form. Participants were also asked to set a quit date agreed upon with the researchers during the initial installation of the App. The quit dates were 3 for each participant, each agreed and discussed with the researchers during the follow-ups. The App sent daily requests via push notifications to participants to ask them to enter the diary entry to report their smoking status. Participant demographics were collected using a standardized form, then participants were invited to describe how much and what they were smoking, if they had used nicotine substitutes in the last 3 months, if they had a smartphone, and if they mastered the English language; finally, any symptoms were evaluated.

Baseline visit (Visit 1): the baseline visit represented the effective start date of the path where we asked the participants to specify again how much they smoked, the level of craving of the cigarette, then the level of exhaled CO (eCO) was measured and the participant agreed with the researcher a first quit date; finally, any symptoms were evaluated.

Between the baseline visit and the final visit, two follow-up visits were made by telephone or email, the first after the first month (week 4) and the second after the second month (week 8). These remote visits had the function of monitoring the process of the study, checking whether the participant had encountered problems or had any doubts of all kinds, and, if the complete cessation of smoking had not yet occurred, establishing a new quit date for the following month as a goal.

Final visit (Visit 2): participants were required to complete the App daily and to attend a final onsite three months after the baseline visit in order to uninstall the App, assess the smoking status from participants self-declarations verified by the exhaled CO measurement and finally do a qualitative semi-structured interview with the participants in order to fully understand feasibility, acceptability, effectiveness, and how they experienced the use of the App; after completing the three months, the following questions were asked to the participants after measuring the eCO: total number of days in which the diary of the app was not completed, if the participant has used nicotine substitutes since the last visit, level of craving, number of “Missions” obtained in the app, total number of times the participant contacted the app’s internal “support”, smoking cessation app utility, app ease of use, app aesthetic appreciation, any suggestions for the app, if the app has worked properly and that smartphone has used, most useful features of the app, most popular features of the app, extra information that the participant would like to see in the app, difficulties in using the app, what the participant would include in the next version of the app, if the app helped it and how, any final comments; finally, any adverse symptoms were evaluated. The term “smoking cessation” in this research pertains to quitting the use of traditional cigarettes that involve combustion.

We did consider an accepted range of days from the planned visits. Specifically, we allowed a range of ± 5 days for the visits. This flexibility in the timeline was taken into account to accommodate any potential scheduling challenges or unforeseen circumstances that participants might encounter.

Data of each subject were collected at each visit and noted in ad-hoc constructed case report forms (CRFs), paper or digital questionnaires used specifically in clinical research.

### Data analysis

Written notes were taken by the researcher during the individual interviews and when the participants were “Thinking-Aloud”; the researcher observed the patient in person or observed their tone and behavior, as in the case of telephone or telematic interviews, e.g., to detect any difficulties encountered. We utilized the coding method of “content analysis” to interpret the meaning of what the participants reported in a quantitative form; as for the “Thinking-Aloud” method, the content of what the participants said was coded through a “narrative analysis”, in combination with qualitative “content analysis”.

The data analysis was informed by the study objective to assess the feasibility and usability of the Smoke Free app. We were interested in the study participants’ views on the information provided in Smoke Free, usability, usefulness as support in smoking cessation paths, perceptions of graphics and text, difficulties with navigating, and overall understanding of the Smoke Free app and perceived relevance for Italian people who smoke. These aspects have been reported to be relevant aspects when assessing feasibility studies of information systems [11].

Quality assurance (QA) was guaranteed through rigorous data handling and analysis procedures at every stage of data collection in which we recorded, transformed, or analyzes data. We also checked for potential errors in data before producing the datasets. QA was also required from the involved Internal Ethic Review Board of Psychology Research (IERB).

Descriptive statistics were used to summarize the results. The repeated measures ANOVA and Wilcoxon paired samples test (2-tailed) were used to test for differences in smoking consumption, self-reported craving level, and measured eCO level between the assessments. All quantitative analyses were performed with the statistical software Jamovi version 2.3.17.0. Interviews were conducted in person, over the phone or via telematics by one researcher and the participants’ responses to the semi-structured interview were noted during the interviews. Data were analyzed according to the principles of qualitative description: specifically, we chose to use the steps of Data Logging, Data Coding and Thematic Networks [12]. The latter were also employed in organizing the answers given by the subjects, related to feedback on user experience, during the final interview.

During these interviews, participants were administered Case Report Forms (visible as Supplementary material), in which the subject’s personal data (obtained with prior informed consent) and various questions divided according to the type of visit were included. The subject was asked to fill out the form on his or her own under the supervision of the researcher, or the researcher reported verbatim the answers given by the subject in the case of an interview conducted by telephone or telematics (or in the case of other issues).

The data obtained were then translated quantitatively to be entered into an Excel spreadsheet and analyzed, either on Excel itself (e.g., for graphing) or on Jamovi.

## Results

### Participant characteristics

Thirty participants enrolled in the study; 28 participants reached the first follow-up visit (at week 4), while 22 completed the protocol reaching the final visit (Visit 2). Table 1 shows the baseline characteristics for all enrolled participants (N = 30). Among all enrolled participants, most were aged between 21 and 54 years (Avg = 31; SD = 10,42), female, and had obtained a degree. Most had tried to quit smoking previously (n = 22), smoked n = 12 cigarettes per day on average at the screening visit and smoked on average for 12 years.

**Table 1:** Baseline characteristics of enrolled participants

	Enrolled participants at baseline N = 30
Age, *Mdn* (IQR)	25 (11)
Sex, *n* (%)	
Male Female	13 (43.33) 17 (56.67)
Education, *n* (%)	
Middle school High school Degree or higher	2 (6.67) 10 (33.33) 18 (60)
Cigarettes smoked per day, *Mdn* (IQR)	10 (9)
Number of years since smoking, *Mdn* (IQR)	9 (8.50)
Cessation attempts, *Mdn* (IQR)	2 (2.75)
eCO, *Mdn* (IQR)	17 (6)

**Fig. 1:**
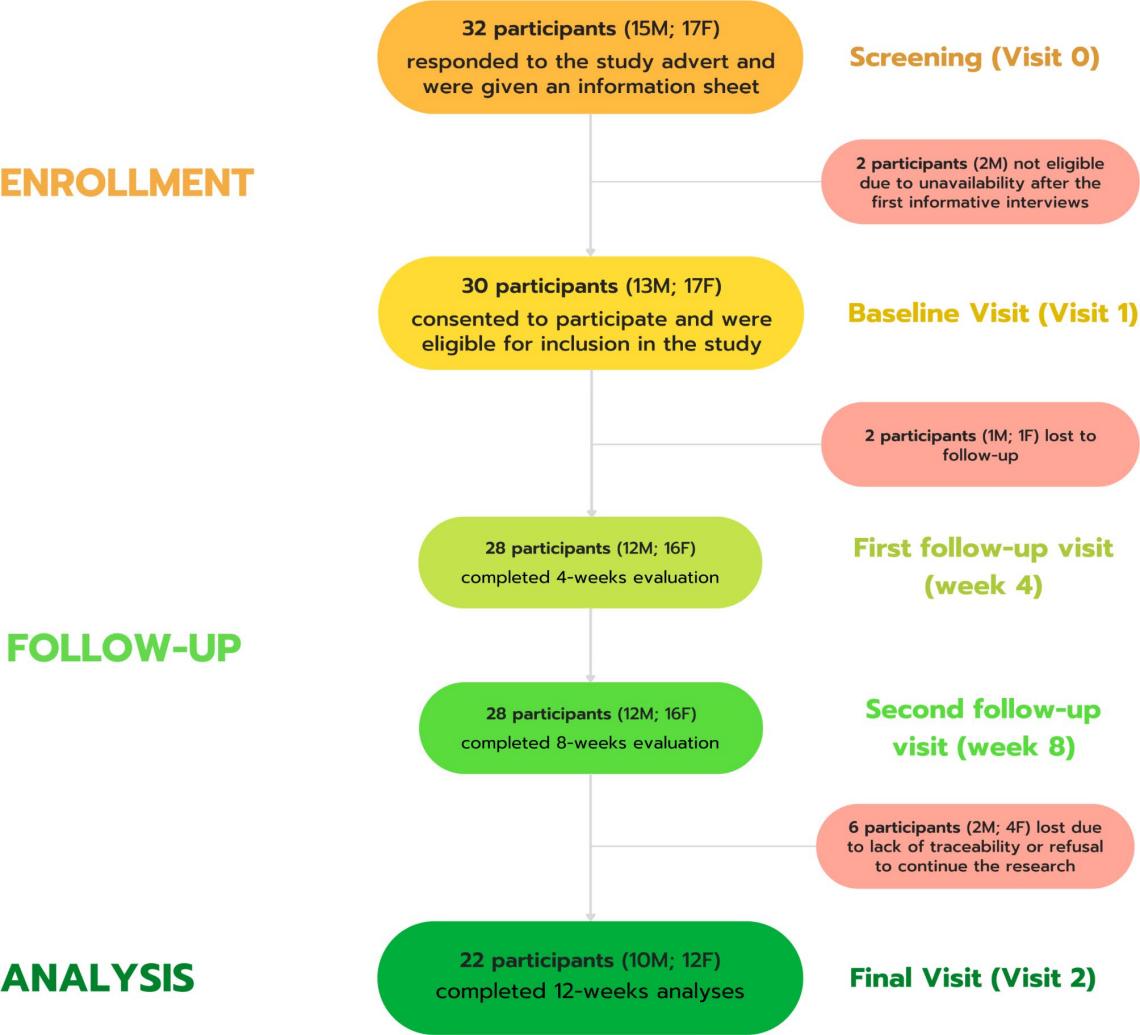
Study flowchart

Participants discontinued their involvement in the study at various time points. Most participants (n = 6) withdrew their participation between the second follow-up visit (week 8) and the final visit (week 12), as depicted in Fig. 1. Two participants (n = 2) were rendered ineligible for the study after the initial informative interviews, exhibiting a cessation of communication with the researcher via calls and emails, and ultimately becoming untraceable without a clear explanation, even foregoing the signing of the informed consent form. Another two participants (n = 2) left after the first follow-up session: one individual expressed interest in leaving the study due to a health issue that led to a cessation of smoking and a desire to capitalize on this state. Conversely, the other participant wished to exit the study since they preferred maintaining their smoking status, having already reduced their cigarette consumption to 1–2 cigarettes per day. Subsequently, six participants were again lost to the study due to untraceability (n = 3) or their refusal to continue participating (n = 3). The latter group’s decision was primarily attributed to their perception of having attained complete smoking cessation (n = 2) or transitioning to the use of electronic cigarettes (n = 1).

Through a comparative descriptive, we can make different observations about the different variables between the participants who completed the follow-up visits (FUP; n = 22) and those who did not complete the follow-up visits (NOFUP; n = 8) (Table 2). In summary, the FUP group tends to have higher means, medians, and generally more variability (higher SD, variance, larger IQR, and wider range) across the variables compared to the NOFUP group. This suggests that participants in the FUP group exhibit greater diversity and potentially more extreme values in these variables than those in the NOFUP group. The broader ranges and wider distributions in the FUP group potentially reflect a richer tapestry of smoking habits, cessation attempts, and smoking-related characteristics.

**Table 2:** Characteristics of enrolled participants who completed the follow-up visits (FUP) and those who did not complete the follow-up visits (NOFUP).

	Mean	Median	SD	Variance	IQR	Range
Age						
FUP	30.91	25.00	10.792	116.468	11.000	33
NOFUP	29.88	25.00	9.978	99.554	6.000	23
**Cig/day (Baseline)**						
FUP	13.32	11.00	8.828	77.942	9.000	35
NOFUP	8.50	6.50	5.099	26.000	5.000	15
**Years of Smoking**						
FUP	13.00	9.00	10.132	102.667	7.250	35
NOFUP	9.38	9.00	5.579	31.125	10.250	14
**Cessation Attempts**						
FUP	3.09	2.00	4.450	19.801	3.750	20
NOFUP	1.13	1.00	0.641	0.411	0.250	2
**eCO (Baseline)**						
FUP	18.05	17.00	7.669	58.807	6.000	32
NOFUP	14.13	13.00	3.682	13.554	2.000	11

### User evaluation of the Smoke Free app’s feasibility

Participants reported that they had not completed the internal diary of the app for an average of 39 days out of the 90 days of the study (Mdn = 49.5; SD = 30.2; IQR = 50.8) and that they had obtained on average 4 Missions related to the objectives (Mdn = 0; SD = 9.12; IQR = 3.75), on the corresponding screen. Only 3 participants said they had contacted Support inside the app, an average of 5 times each (Mdn = 5; SD = 3.51; IQR = 3.50).

Participants rated the following app features on a scale of 1 to 10, as showed in Table 3:

**Table 3:** App features evaluated on a scale of 1 to 10

App Features Evaluated	Average Rating (1–10)
Smoking cessation utility	5.3
User-friendliness	7.5
Aesthetic appeal	8.5

The user interface can be measured as the user’s satisfaction in achieving pragmatic and hedonic goals, and pleasure, thus contributing to a greater investment in the use of the app [13]. This app was evaluated having good aesthetic appeal and user-friendliness but being moderately useful, despite some participants quitting or reducing their smoking behavior.

To improve the app, participants have been interviewed and the following additions have been proposed for future app updates, as seen in Table 4:

**Table 4:** Proposed additions for future updates of the app

Proposed Additions for Future Updates	Participants (%)
Italian language	27.27%
More notifications	18.18%
Improvements to Missions section	4.55%
Increase in motivational factor by the app	4.55%
Evaluation of data entered in the diary	4.55%
Social networks integration	4.55%
Biophysical changes over time due to not smoking	4.55%
Damage caused by smoking over time to the body	4.55%
No further suggestions for future features	27.27%

6 out of 22 participants had no further feature suggestions to add to the app in the future.

The most useful features indicated by participants were the following, showed in Table 5:

**Table 5:** Most useful features indicated by participants related to the app

Most Useful Features Indicated	Participants (%)
Charts	40.91%
Diary	13.64%
Missions	18.18%
Agenda	4.55%
Craving monitoring	4.55%
Monitoring in general	4.55%
Reminders	9.09%
Tips	4.55%

At the end of the study, 5 participants reduced continuously and quit smoking from the first follow-up visit (week 4) to the final visit (Visit 2, week 12), and 9 reduced their daily cigarette smoking consumption of at least 50% compared to baseline.

Finally, participants were asked to indicate if and how the App helped them quit smoking: 10 out of 22 participants said the app didn’t help them quit; 5 out of 22 said the app helped them understand the cost of smoking and the number of cigarettes not smoked; 2 out of 22 said that the app helped them, in particular, to increase their awareness of smoking; 2 out of 22 said that the app helped them, with particular reference to the notification and reminder system; 2 out of 22 said that the app helped them, in particular in the monitoring of smoking; 1 in 22 said that it helped to give him more motivation. Both subjective measures, produced and perceived by the participants (Tables 3 and 4), and objective measures, inherent to the app (Table 5), were analyzed; it is indeed important to distinguish and empirically compare subjective and objective usability measures [14].

Data were collected from patients through the Think-Aloud method. In particular, the collected data provide indications of patients’ difficulties both during the Case Report Form (CRF) compilation process and during the continuation of the research. Out of the total n = 30 instances, n = 16 exhibited struggle behaviors, while the remaining n = 14 did not. The observed prevalence of struggle behaviors indicates potential challenges and complexities encountered by participants while completing the CRFs, shedding light on their decision-making and regulatory processes. On the other hand, no other or more specific difficulties were recorded, e.g., in connection with answering individual questions on the number of cigarettes smoked; we assume that this is motivated by the prerequisite of the motivation to quit.

### Daily smoking consumption

The efficacy of the application in facilitating smoking cessation was also evaluated. Table 6 presents the results of a Paired Samples t-test comparing the baseline (BL) daily cigarette consumption to the daily cigarette consumption at visit 2 (V2). The t-statistic value is 4.60, with 21 degrees of freedom (df). This suggests that there is a significant difference in daily cigarette consumption between baseline and visit 2. Also, the average value of the number of cigarettes smoked per day decreased by approximately 31.73% compared to the average value recorded in the baseline visit. The reduction was found to be statistically significant, as the p-value is less than 0.001, which is statistically significant at the conventional 0.05 level.

**Table 6:** Paired Samples T-Test comparisons between BL and V2 of cigarettes smoked per day

						statistic		df		p	
BL Cig/die		V2 Cig/die		Student’s t		4.60		21.0		< 0.001	

Table 7 displays descriptive statistics for daily cigarette consumption at three time points: Baseline Visit (Visit 1), First Follow-up Visit (4 weeks), and Final Visit (Visit 2, 12 weeks). At Baseline Visit, the mean daily cigarette consumption was 12.03 (SD = 8.21), with a median of 10.00 and an SE of 1.50. At the First Follow-up Visit, the mean decreased to 10.50 (SD = 5.98), with a median of 9.00 and an SE of 1.13. By the Final Visit, the mean further decreased to 8.18 (SD = 9.07), with a median of 5.50 and an SE of 1.93. These statistics suggest a consistent decline in daily cigarette consumption over time, confirmed by repeated measures ANOVA: daily cigarette consumption by participants decreased with a statistically significant difference between daily consumption reported at the baseline visit and that reported at the last visit (p < .001) (Table 8).

**Table 7:** Descriptive table of cigarettes smoked per day across the study

		N		Mean		Median		SD		SE	
Baseline Visit (Visit 1)		30		12.03		10.00		8.21		1.50	
First Follow-up Visit (4 weeks)		28		10.50		9.00		5.98		1.13	
Final Visit (Visit 2, 12 weeks)		22		8.18		5.50		9.07		1.93	

**Table 8:** Repeated Measures ANOVA was used to compare cigarettes smoked per day between Visit 1, the First Follow-up Visit (4 weeks) and Visit 2 (12 weeks). Post Hoc Comparisons of cigarettes smoked per day table. Post Hoc tests help to understand which pairs among the investigated groups exhibit statistically significant differences in daily cigarette consumption

Comparison							
Smoke Free Trial		Smoke Free Trial	Mean Difference	SE	df	t	p_tukey_
Baseline Visit (Visit 1)	-	First Follow-up Visit (4 weeks)	2.36	1.24	21.0	1.91	0.160
	-	Final Visit (Visit 2, 12 weeks)	5.14	1.12	21.0	4.60	< 0.001
First Follow-up Visit (4 weeks)	-	Final Visit (Visit 2, 12 weeks)	2.77	1.31	21.0	2.12	0.109

Table 9 provides a comprehensive descriptive analysis that delves into the comparison of characteristics between two distinct groups of participants during Visit 4, which corresponds to week 12 of the study. The focus of this comparison is on participants who successfully managed to reduce their daily cigarette consumption to a level below 5 and those who did not achieve this reduction. Male participants who smoke more than 5 cigarettes per day have an average age of 35.33 years (SD = 12.09), with a median age of 32 and an IQR of 17 years. Female participants with the same smoking habits are notably younger, with an average age of 23.86 years (SD = 1.57), a median age of 24, and a narrow IQR of 1 year. Regarding smoking history, male smokers in this category have an average duration of 17.5 years (SD = 12.97), while their female counterparts have a shorter average duration of 7.71 years (SD = 2.21). In terms of smoking cessation attempts, male participants have made an average of 6.17 attempts (SD = 7.65), whereas female participants average around 1.14 cessation attempts (SD = 1.57).

**Table 9:** Descriptive overview of the characteristics’ comparison between participants who achieved a daily cigarette consumption of under 5 and those who did not, at Visit 2 (week 12)

	Cig/day Visit 2	Sex	N	Mean	Median	SD	IQR
Age	> 5	Male	6	35.33	32.00	12.09	17.00
		Female	7	23.86	24.00	1.57	1.00
	< 5	Male	4	39.00	38.50	16.19	27.50
		Female	5	29.00	26.00	6.48	8.00
Years Of Smoking	> 5	Male	6	17.50	12.50	12.97	13.75
		Female	7	7.71	8.00	2.21	3.00
	< 5	Male	4	21.50	21.00	12.92	20.00
		Female	5	8.20	7.00	3.96	2.00
Cessation Attempts	> 5	Male	6	6.17	3.00	7.65	7.25
		Female	7	1.14	0.00	1.57	2.00
	< 5	Male	4	3.00	3.00	0.00	0.00
		Female	5	2.20	2.00	2.28	4.00

### Differences in self-assessment and biometric parameters

#### Level of self-perceived craving

The average value of the self-perceived level of craving decreased by 17.09% compared to that recorded in the first follow-up visit. Although the average of the self-perceived craving level has decreased, it should be noted that the standard deviation has slightly increased; this confirms the general decrease in smoking cessation trend of the sample reported in the previous paragraphs and underlines the presence of possible outliers. Finally, the level of self-perceived craving did not decrease statistically significantly from the first follow-up visit to final visit (Table 10).

**Table 10:** Wilcoxon matched-pairs test table of level of self-perceived craving between the first follow-up visit (at 4 weeks) and final visit (Visit 2, 12 weeks)

Comparison			Statistic	p	Mean difference	SE difference
CravingLvlFU1	CravingLvlLV	Wilcoxon W	151 ^a^	0.002	1.50	0.373

Note. H_a_ μ _Measure 1 − Measure 2_ > 0

^a^ 4 pair(s) of values were tied

#### eCO levels

The average value of the eCO level measured at the end of the study was 20.59% lower than that of the baseline visit, indicating a promising cleaning of the participants’ respiratory tract. The difference between the average eCO in baseline visit and last visit (Visit 2, week 12) is statistically significant (p < .001) (Table 11).

**Table 11:** Repeated Measures ANOVA was used to compare eCO levels between Visit 1, the First Follow-up Visit (4 weeks) and Visit 2 (12 weeks). Post Hoc comparisons of eCO measurements table. Post Hoc tests help to understand which pairs among the investigated groups exhibit statistically significant differences in eCO levels

Comparison							
Smoke Free Trial		Smoke Free Trial	Mean Difference	SE	df	t	p_tukey_
Baseline Visit (Visit 1)	-	First Follow-up Visit (4 weeks)	2.09	1.08	21.0	1.93	0.155
	-	Final Visit (Visit 2, 12 weeks)	4.55	1.01	21.0	4.50	< 0.001
First Follow-up Visit (4 weeks)	-	Final Visit (Visit 2, 12 weeks)	2.45	1.27	21.0	1.94	0.152

#### Qualitative findings

As reported in the previous paragraphs, relevant phrases and expressions spoken by the participants were noted down during the interviews. Below we present an example of difficulty in continuing the path of reducing cigarette smoking, while nevertheless indirectly preserving the use of the app:

“In the initial phase, the app was very helpful. I went from about 40 cigarettes a day to an average of 20, reducing to 15 on the best days. This was within the first two weeks of usage.

Then, in the last 20 days, I went through a somewhat complicated period. The children got COVID, followed by the rest of the family, including myself. Amidst the stress of the situation, everything fell apart, including cigarette tracking and motivation. Now, I’m trying to get back on track with the control and reduction attempt. I hope this update is sufficient. […] Of course, I’d like to add that any assistance and input from your side to help me with this challenging endeavor is welcome.”

The app is also mentioned in the following follow-up. The participant found the app useful, considering it effective in reducing the number of cigarettes smoked “when everything is fine”, but identified “complicated periods” as a significant barrier, suggesting the insufficiency of this tool when more challenging contextual factors come into play.

“Unfortunately, I don’t have good news. The app has led me to a general decrease in the number of cigarettes smoked. It does provide greater awareness of the issue, but it doesn’t fully resolve it. […] The app is a good support, but not a definitive solution for cases like mine. It’s a good support tool when everything is going well, but not very effective during complicated periods.“

The feedback regarding the app’s usefulness during stress-free periods and its reduced effectiveness during stressful conditions is also confirmed by other participants:

“I find the app helpful and had reduced cigarettes, but then I faced new family conditions that increased my stress, and now I’m smoking as much as before.“

“Now I manage to stay around an average of 2 cigarettes per day, except for some particularly stressful days.“

“Unfortunately, during periods of high stress, cravings become unbearable, and I end up exceeding.“

Interestingly, for one participant, the frustration of having to use the app also proved to be beneficial:

“I significantly reduced smoking, partly due to the frustration of entering new entries every time I gave in.“

In conclusion, while some participants appreciated and found useful the interactive graphing function for tracking and motivation, there were others who chose to avoid using it because “it was disheartening not to be able to make a significant improvement and have that feedback thrown in their face”.

## Discussion

Good feasibility has emerged regarding the use of the smartphone application “Smoke Free” in a sample of Italian people who smoked.

Participants generally found the app pleasant and functional, with interesting and useful features and suggested more content for future updates. The use of smoke-fighting tools with a pleasant, non-threatening design makes it more likely that the person who smokes will continue the path of cessation, and not feel threatened [15]. Almost all participants have proposed translating the application fully into Italian to further improve its effectiveness in Italian territory. Participants in this study reported in the initial screening interview that they had never made use of smartphone apps specially designed to help with smoking cessation (except for one participant): this could have been an advantage, exploiting the novelty effect introduced using an app [16], or a disadvantage, aligning with a possible trail of pessimism and little confidence in this tool [17].

Two different studies examined the effectiveness of two smoking cessation tools found in the app: “Missions” and the Quit Coach support section. Both studies used a randomized control design, which means that participants were randomly assigned to either a group that used the tool or a control group that did not. The first study, conducted by Crane et al. [18], found that users who used the “Missions” tool were twice as likely to quit smoking compared to those who did not use the tool. “Missions” is a feature within a mobile app called “Smoke Free”, the same used in this study, which provides users with daily tasks and challenges to complete in order to help them quit smoking. The second study, conducted by Perski et al. [19], examined the effectiveness of the Quit Coach support section within the “Quit Genius” app. The study found that the Quit Coach, which provides personalized coaching and support through in-app messaging, further increased quit rates among users.

Out of 22, 12 participants (54.55%) reported that the app helped them in their path of cessation from smoking, also specifying how this was important in motivating them and helping them to monitor their cigarette consumption. Participants also reduced their daily cigarette consumption statistically significantly.

The results of this study reflect the findings of Regmi and colleagues in their review [7], in which they analyzed the following smartphone applications designed for smoking cessation support: Computer-assisted Education system (CO-ED), Real E Quit Mobile application (REQ-Mobile), Smart Quit, SmokeFree28, and ACT-based cessation App. The authors claimed that “the use of such applications seemed to increase cessation rates among people who smoke, although adherence to the internal functionalities of the application appeared to influence cessation rates”; the variable of adherence to the internal functionalities of the app has not been evaluated or correlated, in our study, with the smoking cessation rates of the participants; nevertheless, to make a comparison, our study reports a statistically significant decrease in daily cigarette consumption, but, at the same time, a minimum general adherence of participants to the internal functionality of the app: participants failed to fill out the internal diary of the app for an average of 39 days out of 90, they got an average of 4 Missions and only 3 participants said they contacted the support within the app. Regmi and colleagues then stated that audio-visual features followed by a quit plan, progress tracking, and sharing features were the most liked and used application features; in this study, the progress and current status tracking function was greatly appreciated by the participants, as 9 out of 22 participants indicated that they had found the charts on biometric, health and economic data useful, and 2 other participants said they found the app useful in general for monitoring, both generic (1 out of 22) and specific for craving (1 out of 22).

We acknowledge the potential for our study to inform the development of more tailored digital health solutions for smoking cessation. Specifically, considering the significant influence of stress on smoking behaviors, the Smoke Free app design could in the future incorporate stress management techniques or modules to enhance its effectiveness. For instance, the app could offer relaxation exercises, meditation guides, or even provide links to counseling services, thereby addressing the underlying stressors that often contribute to smoking relapse. Additionally, during perceived “complicated and stressful periods,“ the app could implement more frequent check-ins or motivational messages to sustain user engagement and support individuals in maintaining their commitment to quitting smoking. By integrating these features, Smoke Free could offer a comprehensive and personalized approach to smoking cessation, catering to the unique needs and challenges faced by individuals on their journey towards a *literal* smoke-free life.

This research has several limitations. Firstly, the number of participants is decidedly limited. Further studies will be necessary to validate our findings in our reference sample, preferably with a larger number of participants, to acquire more reliable data and results. Another limitation pertains to the fact that the data upon which this research is based relies on self-reported information from the participants, hence the results and conclusions are not derived from definitive and incontrovertible data, which might introduce potential biases.

Moreover, another limitation of our study is the absence of a control group. Drawing conclusive inferences becomes challenging without comparing the results to those of a group that did not undergo the same manipulation or intervention. The inclusion of a control group is pivotal in minimizing the influence of confounding variables and eliminating alternative explanations for the observed results in our study. Therefore, future research endeavors that incorporate a control group are imperative to confirm and bolster the validity of the findings observed in our sample.

Lastly, a limitation stems from the study’s design. The decision not to adopt a random allocation design was influenced by the preliminary and exploratory nature of the research, designed to be relatively straightforward in terms of recruitment and allocation from the outset, as it lays the groundwork for more complex future studies based on the results obtained (for example, a more comprehensive and intricate study will be conducted with an enhanced version of the application). Furthermore, the inclusion criteria for this study targeted individuals who were uncommon, even rare, to find. For instance, the English language requirement of the app posed a barrier that will need to be overcome in future studies in an Italian context to enhance and facilitate recruitment. Not employing random allocation certainly impacts the study and is a rationale for its implementation in subsequent research.

## Conclusions

The study demonstrated that the Smoke Free app is a promising tool for promoting smoking cessation among Italian people who smoke. The participants found the app user-friendly and effective in providing motivational messages, tracking progress, and offering various resources to support their quit attempt. However, the study also identified some areas for improvement to optimize the App’s impact on smoking cessation rates. One of the primary suggestions from the participants was to add an Italian translation feature to the App, which would make it more accessible and relevant to Italian people who smoke. Additionally, the study found that participants would have appreciated receiving more frequent notifications and reminders to stay on track with their quit attempt. By integrating these recommended features, the Smoke Free App could not only increase its efficacy in supporting smoking cessation but also better cater to the needs and preferences of Italian people who smoke. Overall, this research provides valuable insights into how mobile health interventions such as this App can be tailored to specific populations to improve their effectiveness in promoting behavior change.

### Electronic supplementary material

Below is the link to the electronic supplementary material.

Supplementary Material 1

## Data Availability

The datasets used and/or analysed during the current study are available from the corresponding author on reasonable request.

## Abbreviations

mHealth: Mobile Health
mCessation: Mobile phone-based tobacco cessation
TMQ: Test My Quit
C2Q: Craving to Quit
eCO: Exhaled carbon monoxide
ECs: E-cigarettes
HTPs: Heated Tobacco Products
GDPR: General Data Protection Regulation
IERB: Internal Ethic Review Board of Psychology Research
AIP: Italian Association of Psychology
CO-ED: Computer-assisted Education system
REQ-Mobile: Real E Quit Mobile application
