# Supplementary material for: User evaluation and feasibility test of an app designed for smoking cessation in Italian people who smoke: preliminary findings from an uncontrolled pre-test post-test open study
Source: BMC Psychol. 2023 Nov 10;11:387. doi: 10.1186/s40359-023-01430-w (PMC10636835; doi:10.1186/s40359-023-01430-w)
Supplement: Supplementary file 1 — Supplementary Material 1 [file 40359_2023_1430_MOESM1_ESM.pdf]

REGISTRATION

First and last name:
Address:
Contact details:
Sex:
Age:
Education level:
Patient screening number:
Comments:

Visit date: **SCREENING VISIT 0**

Signed informed consent form ☐	Checking inclusion and exclusion criteria ☐
Number of cigarettes/day (to be enrolled, must be ≥5/day for at least one year):	How many years have you been smoking?
Brand of used cigarettes:	Number of quit attempts:
eCo level measurement (must be ≥7 ppm to be enrolled):	
In the last 3 months, have you used an electronic cigarette, cigars, chewing tobacco, nicotine replacement, varenicline or bupropion?	☐YES ☐ NO
Completed adverse event form	☐
Do you have a smartphone? (to be enrolled, the answer must be yes)	☐YES ☐ NO
Do you use cannabis regularly or occasionally?	☐YES ☐ NO
Do you have a basic knowledge of the English language? (to be enrolled, the answer must be yes)	☐YES ☐ NO

Visit date: **VISIT BL N 1**

Number of cigarettes/day:	eCo:
Quit date (7 to 30 days after visit BL):	Craving level (0 to 10):

Visit date: **VISIT 2**

Completed according to protocol?	☐YES ☐ NO
eCo:	
Number of cigarettes/day:	
Total days on which the internal diary of the App was not compiled:	
Since your previous visit, have you used electronic cigarettes, heated tobacco products (e.g. IQOS or GLO) cigars, chewing tobacco or nicotine substitutes?	☐YES ☐ NO
Craving level (0 to 10):	
Number of total 'Missions' obtained in the App:	
Total number of times you contacted the App's internal 'Support':	
On a scale of 0 to 10, how would you rate the usefulness of this App for quitting smoking?	
On a scale of 0 to 10, how would you rate the user-friendliness of this App?	
On a scale of 0 to 10, how would you rate the aesthetic appeal of this App?	
Suggestions and/or functions to be added in the future to further improve the App:	
Did the App work correctly on your device? And what device did you use during the study?	
Which functions of the App did you find most useful?	
Which elements of the App did you like the most?	
Is there extra information you would like to see on the app?	
What difficulties did you encounter in using the App?	
What would you include in the next version of the App?	
Did the App help you? If so, how?	
Comments:	

End date of study:

Symptoms and Adverse Events (AE) Form:

Ask and check the box for the presence of AE's at each study visit. Report the severity of AEs by using the AE-VAS Scale. Then, report the grade of the severity below the listed AE.

		Visit 0	Visit 1	Visit 2
1 Nausea		☐	☐	☐
2 Vomiting		☐	☐	☐
3 Dry mouth		☐	☐	☐
4 Constipation		☐	☐	☐
5 Diarrea		☐	☐	☐
6 Increase in appetite		☐	☐	☐
7 Anxiety		☐	☐	☐
8 Depression		☐	☐	☐
9 Dyspepsia		☐	☐	☐
10 Palpitations		☐	☐	☐
11 Insomnia		☐	☐	☐
12 Irritability		☐	☐	☐
13 Abnormal dreams		☐	☐	☐
14 Headache		☐	☐	☐
15 Dizziness		☐	☐	☐
16 Fatigue		☐	☐	☐
17 Vertigo		☐	☐	☐
18 Sweating		☐	☐	☐
19 Skin rashes		☐	☐	☐
20 Influenza-like illness		☐	☐	☐
21 Respiratory tract infection		☐	☐	☐
22. Cough		☐	☐	☐

23. Shortness of breath				
24. Throat Irritation		☐	☐	☐
25. Mouth Irritation				
26. Other				
27. …………………………………………………..				
28. …………………………………………………..				
29. …………………………………………………..				
30. …………………………………………………..				

Adverse Event-Visual Analog Scale (AE-VAS)

Mild (Grade 1)	Moderate (Grade 2)	Severe (Grade 3)	Significant (Grade 4)	Serious (Grade 5)

KEY

Mild (Grade 1): Mildly symptomatic, no medical intervention needed
Moderate (Grade 2): Moderately symptomatic, evaluation by PI to determine if medical intervention is necessary.
Severe (Grade 3): Medically significant but not life-threatening cause for an immediate referral for a medical and/or psychiatric evaluation.
Significant (Grade 4): Potentially life threatening, urgent intervention needed.
Death (Grade 5): Results in death; Is life-threatening; Results in inpatient hospitalization or prolongation of existing hospitalization; Results in a persistent or significant disability/incapacity.
